# Gut Bacteria Selectively Altered by Sennoside A Alleviate Type 2 Diabetes and Obesity Traits

**DOI:** 10.1155/2020/2375676

**Published:** 2020-06-25

**Authors:** Zhonghong Wei, Peiliang Shen, Peng Cheng, Yin Lu, Aiyun Wang, Zhiguang Sun

**Affiliations:** ^1^Jiangsu Key Laboratory for Pharmacology and Safety Evaluation of Chinese Materia Medica, School of Pharmacy, Nanjing University of Chinese Medicine, Nanjing 210023, China; ^2^Jiangsu Provincial Second Chinese Medicine Hospital, The Second Affiliated Hospital of Nanjing University of Chinese Medicine, Nanjing 210017, China

## Abstract

Accumulating evidences implicate that gut microbiota play an important role in the onset and prolongation of fat inflammation and diabetes. Sennoside A, the main active ingredient of Rhizoma Rhei (rhubarb), is widely used for constipation as a kind of anthranoid laxative (e.g., senna). Here, we put forward the hypothesis that the structural alteration of gut microbiota in obesity mice may be involved in the pathogenesis of type 2 diabetes (T2D) which may be ameliorated by Sennoside A. We investigated the appearance of obesity, insulin resistance, host inflammation, and leaky gut phenotype with or without Sennoside A in *db*/*db* mice. Horizontal fecal microbiota transplantation (FMT) was used to confirm the critical roles of gut microbiota in the amelioration of the indices in T2D mice after Sennoside A treatment. As a result, we found that Sennoside A administration markedly improved the indices in T2D mice and obesity-related traits including blood glucose level, body weight, lipid metabolism disorder, and insulin resistance. The gut microbiota changed quickly during the onset of T2D in *db*/*db* mice, which confirmed the hypothesis that gut microbiota was involved in the pathogenesis of T2D. Sennoside A altered gut microbial composition which might mediate the antiobesogenic effects in T2D remission. Sennoside A also reduced inflammation and increased tight junction proteins in the ileum in gene-deficient mice via gut microbiota alteration. FMT lowered the blood glucose level and improved insulin resistance, corroborating that Sennoside A perhaps exerted its antiobesogenic effects through gut microbiota alteration. *Chemical Compounds Studied in This Article.* Compounds studied in this article include Sennoside A (PubChem CID: 73111) and metformin hydrochloride (PubChem CID: 14219).

## 1. Background

Obesity, commonly representing an imbalance between energy intake and expenditure, is a metabolic disorder characterized by insulin resistance and *β*-cell dysfunction associated with inflammation caused by overnutrition and other environmental factors [1]. It is strongly correlated with various health problems such as heart disease, stroke, hypertension, and T2D [2, 3]. In the absence of appropriate treatment, T2D resulting from obesity may induce many comorbidities with reduced life expectancy [3, 4].

Trillions of bacteria on the gut are involved in a variety of metabolic functions including fermentation of undigested carbohydrates, modulation of intestinal motility, and synthesis of micronutrients, which have been proposed to play a vital role in the pathophysiology of obesity, T2D, and other metabolic diseases [5]. FMT from the obese human gut can transfer the obese phenotypes to germ-free mice, indicating the causative role of gut microbiota in the development of obesity and metabolic diseases [6]. Therefore, targeting the gut microbiota with appropriate drugs is supposed to be an effective therapy for diabetes, hyperlipidemia, and other metabolic diseases [7]. The inflammation of fat tissue and abnormal fat metabolism induced by gut microbiota disorder are the central mechanism of insulin resistance and diabetes [5, 8]. However, food- and drug-induced T2D animal models are defective because of the secondary action from the food and drugs. Therefore, the *db*/*db* mice which are induced by genetic defects in leptin signaling without any other extrinsic factor are the most suitable mouse model for assessing gut microbiota changes in the progress of obesity and T2D [9].

Rhizoma Rhei (rhubarb), a commonly used herbal medicine, is often used as a laxative and thus is frequently used by obese individuals [10]. Many over-the-counter laxatives consisting of Chinese medicines, such as *Dahuang Tongbian* granule [11, 12] (or rhubarb extract granule for facilitating bowel movement) and *Paidu Qingzhi* capsule [13] (or fat and toxicity reduction capsule), contain rhubarb as well. Sennoside A, an inactive glycoside in rhubarb, is the major purgative component of the herb [10, 14]. Its purgative effect relies on intestinal bacteria which transform Sennoside A into an active metabolite, rhein anthrone [10, 15]. However, its role in keeping slim and improving T2D-related disorders is still unclear. Growing evidences support that rhubarb can alter intestinal bacteria composition and hence exert multiple pharmacological effects [16–18]. Nevertheless, it remains unknown whether Sennoside A affects blood glucose and T2D-related disorders via gut microbiota.

To test the hypothesis that gut microbiota alteration may be involved in the pathogenesis of T2D and may be ameliorated by Sennoside A, the *db*/*db* mice were adopted. The indices of T2D, tissue inflammation, and lipid metabolism were assessed after Sennoside A administration. Also, the effect of Sennoside A on intestinal microbiota was evaluated by analyzing the V3-V4 regions of the 16S rRNA genes by Illumina sequencing and multivariate statistical analysis.

## 2. Materials and Methods

### 2.1. Animal Studies

The animal protocols for the use of mice in this study were approved by the Institutional Animal Care and Use Committees of Nanjing University of Chinese Medicine and followed guidelines issued by the National Institutes of Health. Four-week-old male *db*/*db* mice and male C57BL/Ks mice (wild-type) (*n* = 10) were purchased from the Model Animal Research Center of Nanjing University (Nanjing, China). They were maintained with free access to pellet food and water in plastic cages at 21 ± 2°C and kept in a 12 h light/dark cycle. All efforts were made to reduce the number of animals used and to minimize animals' suffering. Each group of mice was administered daily with either water, 280 mg·kg^−1^ metformin, or Sennoside A at 25 mg·kg^−1^ and 50 mg·kg^−1^ by intragastric gavage for 12 weeks.

### 2.2. Antibiotic Treatment

Antibiotic treatment was performed based on an established protocol [19]. Mice were treated with an antibiotic cocktail of metronidazole (0.75 g/liter), vancomycin (0.5 g/liter), and streptomycin (2 g/liter) in their drinking water for 1 week.

### 2.3. Body Composition

Body weight, liver, and epididymal adipose tissues were measured using a laboratory scale (BT323S Series, Sartorius, China).

### 2.4. Gut Microbiota Analysis by 16S Sequencing

Snap-frozen stool samples (*n* = 5 per group) in liquid nitrogen were stored at -80°C. Total genomic DNA was extracted from samples using the CTAB/SDS method. DNA concentration and purity were monitored by 1% agarose gel electrophoresis. According to the concentration, DNA was diluted to 1 ng/*μ*L using sterile water. Subsequently, 16S rRNA genes were amplified using a specific primer with the barcode. All PCR reactions were carried out in a 30 *μ*L system comprising 15 *μ*L of Phusion High-Fidelity PCR Master Mix (New England Biolabs), 0.2 *μ*M of forward and reverse primers, and about 10 ng template DNA. The thermal cycling consisted of initial denaturation at 95°C for 3 min, followed by 25 cycles of denaturation at 95°C for 30 s, annealing at 55°C for 30 s, and elongation at 72°C for 30 s, and finally at 16°C for 2 min. The composite primer pairs (V3+V4) consisted of the forward primer (5′-CCTACGGGNGGCWGCAG-3′), a composite of 454 primer B and universal bacterial primer 341F, and the reverse primer (5′-GACTACHVGGGTATCTAATCC-3′), a composite of 454 primer A and broad-range bacterial primer 805R. The same volumes of 1x loading buffer (containing SYB green) and PCR products were mixed, and electrophoresis was performed on the samples in 2% agarose gel. Samples with a bright main strip between 460 bp (V3+V4) were chosen for further experiments. PCR products were mixed in equidensity ratios. Then, the mixture of PCR products was purified with a GeneJET Gel Extraction Kit (Thermo Fisher Scientific, USA). Sequencing libraries were generated using a NEBNext Ultra DNA Library Prep Kit for Illumina (NEB, USA) following the manufacturer's recommendations, and index codes were added. The library quality was assessed using the Qubit® 2.0 Fluorometer (Life Technologies, CA, USA) and an Agilent 2100 Bioanalyzer system. At last, the library was sequenced on an Illumina MiSeq platform and 250 bp paired-end reads were generated.

### 2.5. Measurement of Homeostatic Model Assessment-Insulin Resistance Index

Fasting blood glucose (FBG) was measured using glucometer strips (Johnson & Johnson Medical Devices, Hong Kong) that had been done before [20].

Fasting insulin (FI) was measured using the commercial Insulin Mouse ELISA Kit (Cat. No. EMINS, Thermo Fisher Scientific, USA). HOMA-IR was calculated using the following equation: HOMA‒IR = fasting glucose (mg/dL) × fasting insulin (*μ*U/mL)/405.

### 2.6. Fecal Transplantation

Fecal transplantation was performed based on an established protocol [19]. Briefly, 8-week-old male donor mice were administered with NS, metformin, and Sennoside A (50 mg/kg) for 3 months. After 4 weeks of feeding, stools were collected daily for the subsequent 2 months under a laminar flow hood in sterile conditions. Stools from the donor mice of each group were pooled and 100 mg was resuspended in 1 ml of sterile saline. The solution was vigorously mixed for 10 s using a benchtop vortex (MS 3, IKA, Germany) and centrifuged at 800 × g for 3 min. The supernatant was collected and used as the transplant material as described below. Fresh transplant material was prepared on the same day of transplantation within 10 min before oral gavage to prevent changes in bacterial composition. Before being sacrificed for subsequent analyses, 8-week-old male recipient mice (*n* = 5) were inoculated daily with fresh transplant material (100 *μ*L for each mouse) by oral gavage for 8 weeks. Microbiota transplanted mice were kept in an autoclaved cage covered with autoclaved padding and lived with autoclaved feed and drinking water.

### 2.7. Biochemical Analyses

#### 2.7.1. Total Cholesterol, Triglyceride, and FFA Levels

Total cholesterol, triglyceride, and FFA levels were determined by enzymatic colorimetric assays (Jiancheng Bioengineering Institute, Nanjing, China).

#### 2.7.2. TNF-*α* Level

TNF-*α* level was measured using an ELISA specific kit (Ready-SET-Go, eBioscience, USA).

#### 2.7.3. Serum Endotoxin

Serum endotoxin was quantified using a *Limulus* amaebocyte lysate (LAL) kit (Cambrex Bio Science, USA) according to the manufacturer's instructions. Recovery rate was determined based on the net lipopolysaccharide (LPS) concentration of spiked samples supplemented with LPS (0.1 EU/mL, Sigma-Aldrich, USA).

### 2.8. Histomorphological Procedures

Paraffin-embedded adipose sections (6 *μ*m thick) were analyzed after hematoxylin and eosin staining. Five visual fields were randomly observed at 20x magnification by a quantitative pathology imaging system (Mantra, PerkinElmer). Adipocyte quantification was performed using the ImageJ software (http://rsbweb.nih.gov/ij/) for approximately 300-400 cells per mouse (5 animals per group).

### 2.9. Adipose Tissue Fractionation

Adipose tissue was fractionated as described previously [21]. Briefly, the epididymal adipose tissues of different groups were digested with type I collagenase buffer and filtered through a nylon mesh. After centrifugation, fractions of floating adipocytes and pelleted stromal vascular cells were washed several times with PBS and then used for RNA extraction.

### 2.10. Quantitative RT-PCR

Total RNA was extracted from the liver and epididymal adipose tissues using an RNase mini kit (TransGen Biotech, Inc., Beijing, China) and reverse transcribed (Vazyme Biotech Co., Ltd., Nanjing, China). Primers were synthesized by Sangon Biotech (Shanghai, China), and their sequences are provided in Supplementary Table 1. Real-time PCR was performed using SYBR Green PCR Master Mix (TransGen Biotech, Inc., Beijing, China, Beijing, China) and a 7500 Real-Time PCR System (Thermo Fisher Scientific, New York, USA) according to the manufacturer's protocol.

### 2.11. In Vivo Intestinal Permeability Assay [22, 23]

Fluorescence detection in living, anesthetized mice was captured with an IVIS™ live imaging system (IVIS Lumina III, PerkinElmer). Mice were gavaged with fluorescein-isothiocyanate- (FITC-) dextran (4 kDa; Sigma-Aldrich) at a dosage of 400 mg/kg, and then animals were studied 1 hr later using multispectral fluorescent capture. Blood samples were obtained after 4-5 hr by retroorbital bleeding, and the fluorescence intensity in the serum was measured at an excitation wavelength of 485 nm and an emission wavelength of 520 nm using a microplate system (EnSpire, PerkinElmer). FITC-dextran diluted in PBS was used to plot a standard curve, and the serum concentration of FITC-dextran was calculated.

### 2.12. Immunofluorescence Staining and Microscopy

The mouse ileal tissue sections were fixed with Carnoy's fluid and embedded in paraffin. The tissue sections were immunostained with specific antibodies (occludin, Thermo Fisher Scientific, Cat #71-1500; ZO-1, CST Cat #13663) by incubating overnight at 4°C. Following antibody incubation, slides were incubated with Alexa Fluor 594- or 488-conjugated secondary antibody for 2 hr at room temperature followed by washing three times with PBS (3 cycles, 5 min). The nuclei were stained with DAPI and slides were mounted in the ProLong Antifade Reagent. All images were acquired using an inverted fluorescence microscope (Axio Vert.A1, Zeiss).

### 2.13. Western Blot Analysis

One hundred mg of adipose, liver, or intestine tissue was homogenized in a commercial PRO-PREP Protein Extraction Solution (Beyotime, China). Total protein lysates were fractionated by 10% SDS-PAGE and electroblotted onto polyvinylidene difluoride membranes (Immobilon™-P; Merck Millipore, USA). Afterwards, the membranes were blocked with 5% nonfat milk for 1 h at room temperature in TBST buffer (10 mM Tris, 150 mM NaCl, pH 7.6, and 0.1% Tween 20) and probed with primary antibodies overnight at 4°C. The membranes were then incubated with horseradish peroxidase-conjugated secondary antibody. The dilutions of primary and secondary antibodies have been described in Section 2.12. Protein bands were developed using an enhanced chemiluminescence reagent (Merck Millipore). The blots were probed with the primary antibodies against GAPDH (Bioworld Technology, Inc., China), TLR4, IKB-*α*, NF-*κ*B (Abcam, USA), AKT, phospho- (p-) AKT (Ser473), GSK3*β*, and p-GSK3*β* (Ser9) (Cell Signaling Technology, USA).

### 2.14. Statistical Analysis

Unless otherwise stated in the individual subsections of Materials and Methods, statistical analysis was performed using GraphPad Prism V.7.0a. The results represented data from multiple independent experiments. All data were expressed as mean ± standard deviation (SD). The data were analyzed using two-tailed Student's *t*-test (for two groups) and one-way analysis of variance (for more than two groups). Statistically significant differences are shown with asterisks as follows: ^∗^*p* < 0.05, ^∗∗^*p* < 0.01, and ^∗∗∗^*p* < 0.001. Bacterial genuses with a statistically significant difference were assessed using the linear discriminant analysis effect size (LEfSe) method [24] (http://huttenhower.sph.harvard.edu/galaxy). Spearman's correlation coefficients between bacterial genuses and obesity traits were determined by heat map generated using *R* Studio.

## 3. Results

### 3.1. Sennoside A Improved Glucose Homeostasis and Reduced Metabolic Disorders in *db*/*db* Mice

**Figure 1:**
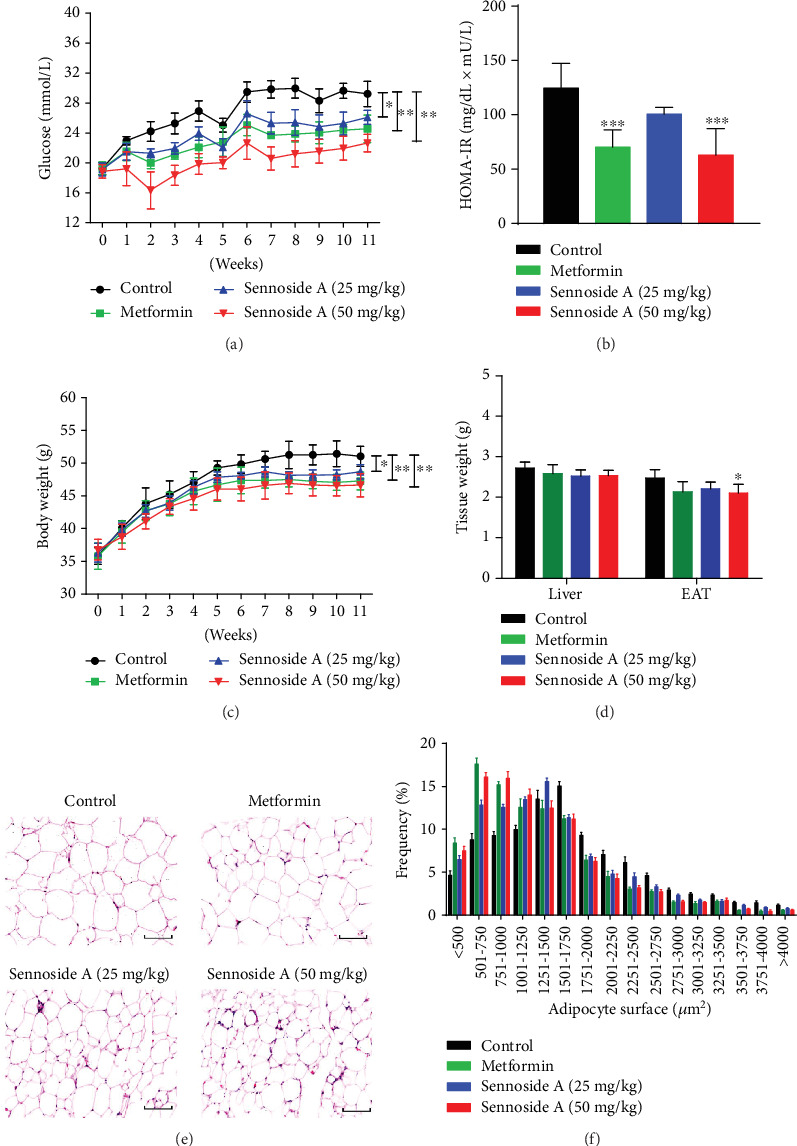
Sennoside A reduced the blood glucose level and fat accumulation in *db*/*db* mice. (a) Glucose level, (b) HOMA-IR, (c) body weight, and (d) tissue weight of *db*/*db* mice during the 12 weeks with or without Sennoside A treatment. (e) Representative hematoxylin and eosin-stained pictures of EAT deposits (*n* = 5 images per mouse). Scale bar: 200 *μ*m. (f) Adipocyte size distribution determined from histomorphometry and image analysis of EAT. Data were presented as means ± SD (*n* = 10 mice per group). Statistical analysis was performed using one-way analysis of variance followed by Kruskal-Wallis test. Significance: ^∗^*p* < 0.05, ^∗∗^*p* < 0.01, and ^∗∗∗^*p* < 0.001 versus control.

Intragastric administration of Sennoside A or metformin (as a positive control drug) [25] for 12 weeks significantly decreased blood glucose, HOMA-IR index, body weight, FBG, body weight gain, TG, TC, and FFA of *db*/*db* mice with T2D [26] compared with those of control mice (Figures 1(a)–1(c) and Figures S1A–S1D). Besides, only accumulation in the epididymal adipose tissue (EAT) decreased, and the liver weight of Sennoside A-treated mice hardly changed (Figure 1(d)). However, the sizes of adipose cells in mice treated with Sennoside A or metformin were smaller (Figures 1(e)–1(f)). Taken together, Sennoside A reduced the blood glucose level and fat accumulation in *db*/*db* mice with T2D.

As a characteristic of adiposity, lipid metabolism was uncontrollable in *db*/*db* mice. Fat accumulation results from an imbalance between the expressions of the genes involved in lipogenesis and lipid oxidation [21, 27]. We performed quantitative PCR for RNAs extracted from the liver and adipose tissues of *db*/*db* mice and control mice to determine the relative mRNA expressions of the genes participating in lipogenesis and lipid oxidation. Sennoside A significantly affected the expressions of these genes in the liver and adipose tissues. Sennoside A could thus reduce fat accumulation by inhibiting the expressions of adipogenic genes *Srebf1*, *Fasn*, and *Scd1* (Figures S2A–S2C) and increasing those of lipid oxidation genes *Acox1*, *Acox2*, and *Cpt1a* (Figures S2D–S2F) in *db*/*db* mice [21].

### 3.2. Composition of Gut Microbiota Changed Quickly in *db*/*db* Mice

**Figure 2:**
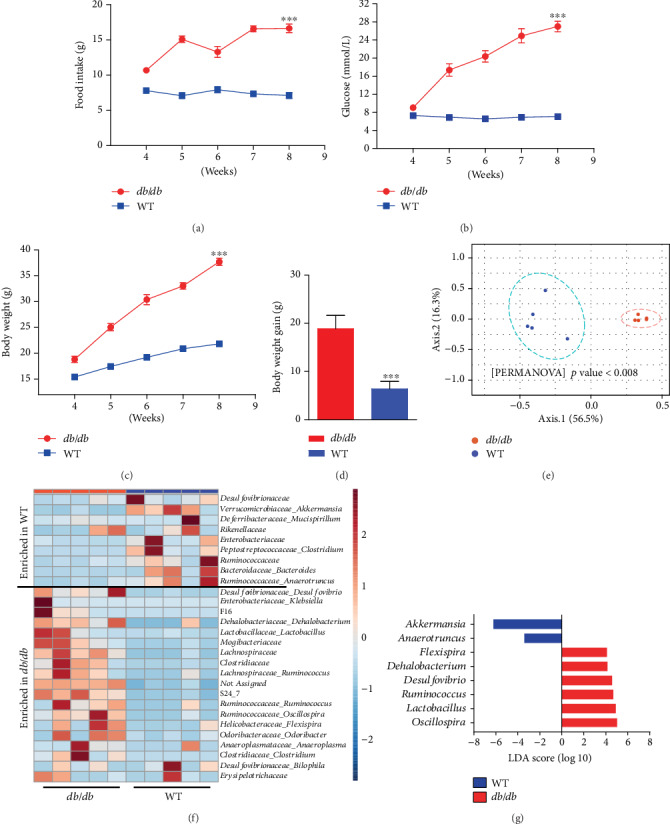
Composition of gut microbiota changed quickly in *db*/*db* mice. Glucose level and body weight rose quickly in 4- to 8-week-old *db*/*db* mice. (a) Food intake, (b) glucose level, (c) body weight, and (d) body weight gain were measured in 4- to 8-week-old mice. (e) Plots were generated using the weighted version of UniFrac-based PCoA with the PERMANOVA significance test. (f) Heatmap showed the relative abundance of prepended higher taxon genuses (19 enriched and 9 decreased ones in *db*/*db* mice). (g) The analysis of differences in the microbial taxa between *db*/*db* mice and WT animals using LEfSe (linear discriminant analysis (LDA) coupled with effect size measurements). Only bacterial taxa reaching a LDA threshold of 2 with a *p* value < 0.05 are shown.

Homozygous mice with spontaneous mutation (Lepr*^db^*) became identifiably obese in the 4th week [28]. Plasma insulin began to increase on the 10th-14th days, so did blood glucose during the 4th-8th weeks. The food intake increased quickly in 4- to 8-week-old Lepr^*db*/*db*^ mice (Figure 2(a)). Meanwhile, the blood glucose level, body weight, and body weight gain also rose evidently (Figures 2(b)–2(d)). Next, we performed a pyrosequencing-based analysis of bacterial 16S rRNA (V3–V4 regions) for fecal microbiota. After removal of unqualified sequences (Materials and Methods), a total of 82,754 raw reads and an average of 38, 384 ± 454 reads per sample were obtained. Then, 37,098 effective reads were generated by rarefaction analysis and the Shannon diversity index, indicating that the sequencing depth covered rare new phylotypes and most of the diversity.

Principal coordinate analysis (PCoA) based on the weighted UniFrac distance matrix revealed a distinct clustering of microbiota composition for each treatment group. The 8-week-old *db*/*db* and wild-type (WT) mice also had significant differences (Figure 2(e)). Notably, taxonomic profiling demonstrated that 8-week-old control mice had similar Firmicutes-Bacteroidetes ratios but had increased relative abundance of Verrucomicrobia and Proteobacteria compared with *db*/*db* mice (Figure S3A) [29]. Furthermore, the relative abundance of prepended higher taxon genus, which was clustered by an averaging algorithm, was expressed as heatmap visualization, including 19 enriched and 9 decreased ones in *db*/*db* mice (Figures 2(f) and 2(c)). In addition, we used the linear discriminant analysis (LDA) effect size (LEfSe) [30] method to identify the most differentially abundant taxons and OTUs (*p* < 0.05 and LDA score > 2) between WT and *db*/*db* mice. Relative to WT mice, *Oscillospira*, *Lactobacillus*, *Ruminococcus*, *Desulfovibrio*, *Dehalobacterium*, and *Flexispira* were more highly abundant and *Akkermansia* and *Anaerotruncus* were markedly decreased in *db*/*db* mice.

To further confirm the hypothesis that T2D was exacerbated by the microbiota, antibiotic cocktail therapy was used. The rapid increases in food intake, blood glucose, and body weight were delayed after antibiotic cocktail therapy as expected (Figure S3B). In short, the gut microbiota in *db*/*db* mice varied significantly, resulting in a composition difference from that of normal healthy mice.

### 3.3. Sennoside A Reversed Gut Dysbiosis in *db*/*db* Mice

Given the contribution of gut microbiota on the development of obesity and metabolic disorders, we studied the effects of Sennoside A on gut microbiota composition by pyrosequencing-based analysis of bacterial 16S rRNA genes in feces. After the removal of unqualified sequences (Materials and Methods), a total of 86,484 raw reads and an average of 81, 668 ± 4,816 reads per sample were obtained. A total of 40,834 effective reads were generated and each fecal sample (*n* = 5) produced an average of 40, 677 ± 452 effective reads. Rarefaction analyses and the Shannon index indicated that the sequencing depth covered rare new phylotypes and most of the diversity.

**Figure 3:**
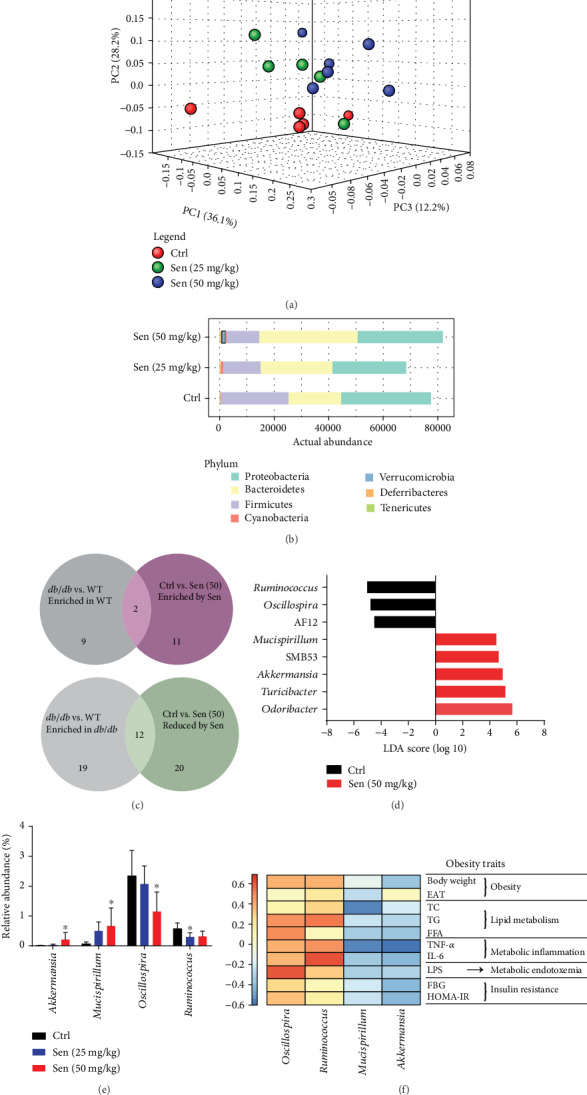
Sennoside A reversed gut dysbiosis of *db*/*db* mice. (a) Principal-coordinate analysis based on the weighted UniFrac index with the PERMANOVA significance test. (b) The relative abundance of microbial taxa was determined on the phylum level of which abundances of >1% were shown as representative. (c) Venn diagram illustrated the number of bacterial OTUs that were differentially abundant between the indicated mice. See also Figure S3C. (d) Linear discriminant analysis (LDA) scores derived from LEfSe analysis, showing the biomarker taxa on the genus level (LDA score) of >4 and a significance with *p* < 0.05 determined by the Wilcoxon signed-rank test. (e) The relative abundance of bacteria genus by different doses of Sennoside A whose levels were altered in the same direction in Sen vs. Ctrl and WT vs. *db*/*db*. (f) Spearman's correlation analysis between the 4 Sennoside A-shifted genus and obesity traits. False discovery rate correction for multiple testing was used.

Weighted UniFrac-based PCoA revealed a distinct clustering of microbiota composition for each treatment group. Permutational multivariate analysis of variance (PERMANOVA) test indicated statistically significant differences in gut microbiota between control *db*/*db* mice and Sennoside A-treated *db*/*db* mice (25 and 50 mg/kg) (Figure 3(a)). Notably, taxonomic profiling demonstrated that Sennoside A increased the Firmicutes-Bacteroidetes ratio and the relative abundance of *Verrucomicrobia* compared with untreated *db*/*db* mice (Ctrl) (Figure 3(b)). We used a heat map to show the 31 genuses of bacteria altered by 50 mg/kg Sennoside A (Sen) in *db*/*db* mice (Figure S3C; Sen vs. Ctrl, 11 increased genuses and 20 reduced genuses in Figure 3(c)).

We sought to identify the Sennoside A-shifted bacterial genuses whose abundance was congruously altered in both Sen versus Ctrl and WT versus *db*/*db*. Detailed analysis showed that 14 genuses were modulated in the same direction, including 2 genuses whose levels were enriched in the Sennoside A and WT group and 12 genuses whose levels were reduced in Sennoside A-treated mice and enriched in *db*/*db* mice (Figure 3(c) and supplementary dataset S1). Consequently, Sennoside A treatment could amplify the relative abundance of “good bugs” (the 2 genuses) in contrast to lower levels of “bad bugs” (12 genuses) in *db*/*db* mice.

Furthermore, LEfSe that was used to identify the most differentially abundant genuses (LDA score > 4, *p* < 0.05) between Sen (50 mg/kg) and Ctrl revealed significantly increased levels of bacterial genuses in the Sennoside A group, such as *Odoribacter*, *Turicibacter*, *Akkermansia*, SMB53, and *Mucispirillum*, together with decreases in AF12, *Oscillospira*, and *Ruminococcus* (Figure 3(d)).

Among the altered genuses identified, *Akkermansia*, *Mucispirillum*, *Oscillospira*, and *Ruminococcus* significantly represented Sennoside A-shifted bacteria whose levels were altered in the same direction in Sen *vs.* Ctrl and WT *vs. db*/*db*. In addition, the relative abundance of these genuses were further validated by different doses of Sennoside A (Figure 3(e)).

We examined whether obesity and T2D-related traits were correlated with the levels of four bacterial genuses effected by Sennoside A. Spearman's correlation analysis showed that levels of these bacteria were correlated with relevant traits. Notably, among these bacteria, *Akkermansia* and *Mucispirillum* (special good bugs) were highly enriched by Sennoside A treatment (Figure 3(d) and Figure S3C) and the two genuses were negatively correlated with all obesity and T2D traits (Figure 3(f)). The levels of *Oscillospira* and *Ruminococcus* (special bad bugs) were negatively associated with obesity and T2D characteristics (Figure 3(f)). These results suggested that the increase of special good bugs and the decrease of special bad bugs might mediate the antiobesogenic effects of Sennoside A during T2D remission.

### 3.4. Sennoside A Relieved Inflammation in *db*/*db* Mice

**Figure 4:**
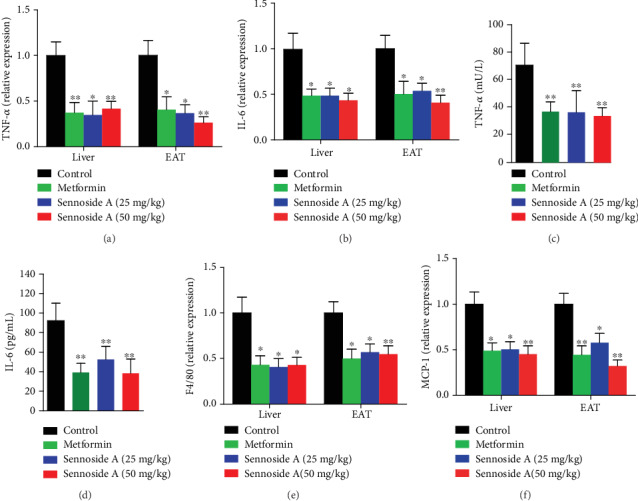
Sennoside A reduced proinflammatory genes and cytokines in the liver and EAT. Relative mRNA expression levels of proinflammatory genes (a) *Tnf* and (b) *Il6* in the liver and EAT. Serum cytokines (c) and TNF-*α* (d) of *db*/*db* mice with or without Sennoside A treatment after 12 weeks. Relative mRNA expression levels of (e) *Adgre1* and (f) *Ccl2* in the liver and EAT (*n* = 5).

T2D mice usually present higher levels of inflammatory cytokines including TNF-*α* and IL-6 in hepatic and adipose tissues [31]. We measured the mRNA expressions of these cytokines after 8 weeks of Sennoside A feeding. *Tnf* (Figure 4(a)) and *Il6* (Figure 4(b)) expression levels were lower in the hepatic and adipose tissues in Sennoside A-treated mice. Moreover, Sennoside A reduced serum levels of secreted *Tnf* and *Il6* in *db*/*db* mice as metformin did [32] (Figures 4(c) and 4(d)).

Obesity is characterized by the infiltration and activation of immune cells in hepatic and adipose tissues. M1 macrophages, which are recruited by monocyte chemoattractant protein-1 (MCP-1), have been proven to be associated with chronic, low-grade inflammation in the adipose tissues of obese animals [33, 34]. We also detected the mRNA expressions of chemokine (C-C motif) ligand 2 (*Ccl2*) encoding MCP-1 and mouse adhesion G protein-coupled receptor E1 (*Adgre1*) encoding epidermal growth factor- (EGF-) like module-containing mucin-like hormone receptor-like 1 (F4/80) in the liver and adipose tissues [35, 36]. Both *Ccl2* and *Adgre1* expression levels were reduced by Sennoside A or metformin treatment compared with those of control mice (Figures 4(e) and 4(f)). Thus, both Sennoside A and metformin relieved inflammation in the liver and adipose tissues in *db*/*db* mice.

### 3.5. Sennoside A Prevented Metabolic Endotoxemia and Leaky Gut in *db*/*db* Mice

**Figure 5:**
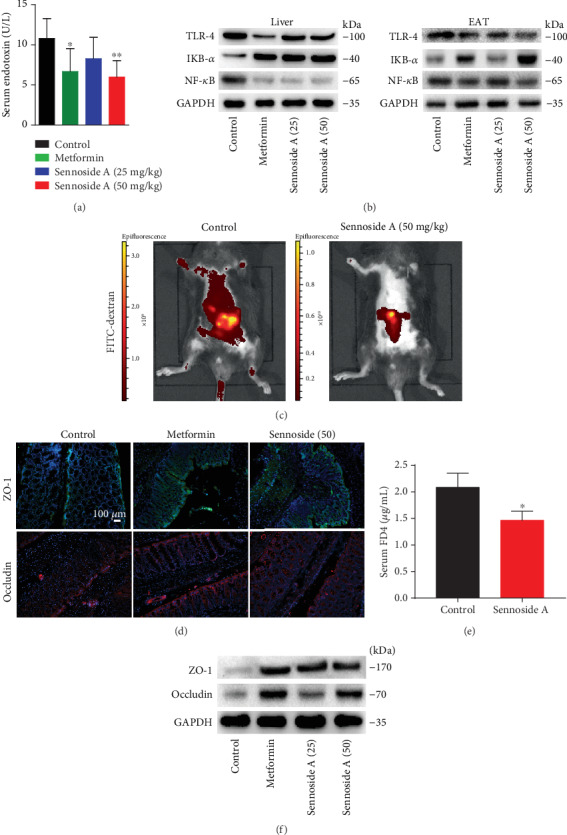
Sennoside A improved intestinal integrity and metabolic endotoxemia in *db*/*db* mice. Serum endotoxin (a) and intestinal permeability (c and e) were measured as described in Materials and Methods. Effects of Sennoside A treatment on TLR4, IkB-*α*, and NF-*κ*B in the liver and EAT (b). Sennoside A prevented expressions and corrected distribution of colonic tight junction proteins, occludin, and ZO-1 (scale bar: 100 *μ*m) (d). Western blot results were consistent with immunofluorescence results, showing a dose-dependent inhibition of expressions of the tight junction proteins in colon tissues (f).

Endotoxemia and TLR4 signaling control the production of proinflammatory cytokines in target tissues [37] and lead to chronic inflammation and insulin resistance in fat mice. Therefore, we evaluated the effects of Sennoside A on serum LPS level (i.e., metabolic endotoxemia) and TLR4 protein expressions in hepatic and adipose tissues. Sennoside A reduced endotoxemia and TLR4 protein expressions in *db*/*db* mice compared with those of control ones (Figures 5(a) and 5(b)). In addition, TLR2 expression and functional activation are increased in recently diagnosed type 2 diabetes, contributing to the proinflammatory state [38]. We evaluated the protein level of TRL2 which is reduced by Sennoside A (Figures S4D and S4E). Since TLR4 signaling pathways can induce the production of proinflammatory cytokines by modulating the activity of NF-*κ*B [39], we examined whether the pathways were affected by Sennoside A supplementation. As shown in Figure 5(b), the production of IkB-*α* interacting with NF-*κ*B prevented its translocation and activation, which was enhanced by Sennoside A treatment. Moreover, Sennoside A decreased NF-*κ*B in hepatic and adipose tissues in *db*/*db* mice (Figure 5(b) and Figures S4A and S4B). Given that the enhanced activation of NF-*κ*B pathways could induce insulin resistance by the dephosphorylation of Akt and GSK-3*β* [40], we assessed the effects of Sennoside A on the related pathways. The phosphorylation of Akt and GSK-3*β* was enhanced by Sennoside A in hepatic and adipose tissues (Figures S4D and S4E). We have also evaluated the activation level of JNK which is activated by inflammatory cytokines and free fatty acids in *db*/*db* mice [41]. JNK activation in both the liver and epididymal adipose tissues was significantly reduced by receiving treatment with Sennoside A (Figures S4D and S4E). Collectively, Sennoside A alleviated endotoxemia and prevented insulin resistance in *db*/*db* mice.

Previous studies have identified a close association between the intestinal barrier dysfunction and metabolic diseases including obesity and T2D [42]. Considering that intestinal dysbiosis in obese animals could affect gut permeability and could subsequently lead to the translocation of bacterial LPS into the blood with the resulting inflammation and insulin resistance [43], we examined whether Sennoside A modulated gut integrity. Firstly, we measured intestinal permeability with FITC-labeled dextran and found that fluorescence in the intestinal lumen was diminished (due to increased absorption of the usually impermeable tracer) in *db*/*db* mice. Live mice imaging after oral administration of FD4 (FITC dextran 4 kDa) revealed that Sennoside A ameliorated the disruption of intestinal barrier integrity in *db*/*db* mice compared with controls (Figure 5(c)). In a confirmatory assay, circulating concentrations of FD4 after gavage were significantly lowered by Sennoside A in *db*/*db* mice (Figure 5(e)). Consistent with decreased access of intestinal contents to the systemic circulation, serum levels of endotoxin, TNF-*α*, and IL-6 were lessened in Sennoside A-treated *db*/*db* mice (Figure 1). Furthermore, Sennoside A reversed the reductions in expressions of zonula occludens-1 (ZO-1) and occludin [23] (Figures 5(d) and 5(f)). These findings suggested that Sennoside A could improve the intestinal barrier integrity in *db*/*db* mice.

### 3.6. Fecal Transplantation of Sennoside A Alleviated Obesity and T2D-Related Traits

The modulatory effects of gut microbiota on T2D-related traits can be transferred to other animals [44, 45]. To determine whether the gut microbiota of Sennoside A-treated animals could improve the insulin sensitivity of *db*/*db* mice, we transferred the microbiota of the treated mice to control *db*/*db* mice and detected their T2D-related traits.

**Figure 6:**
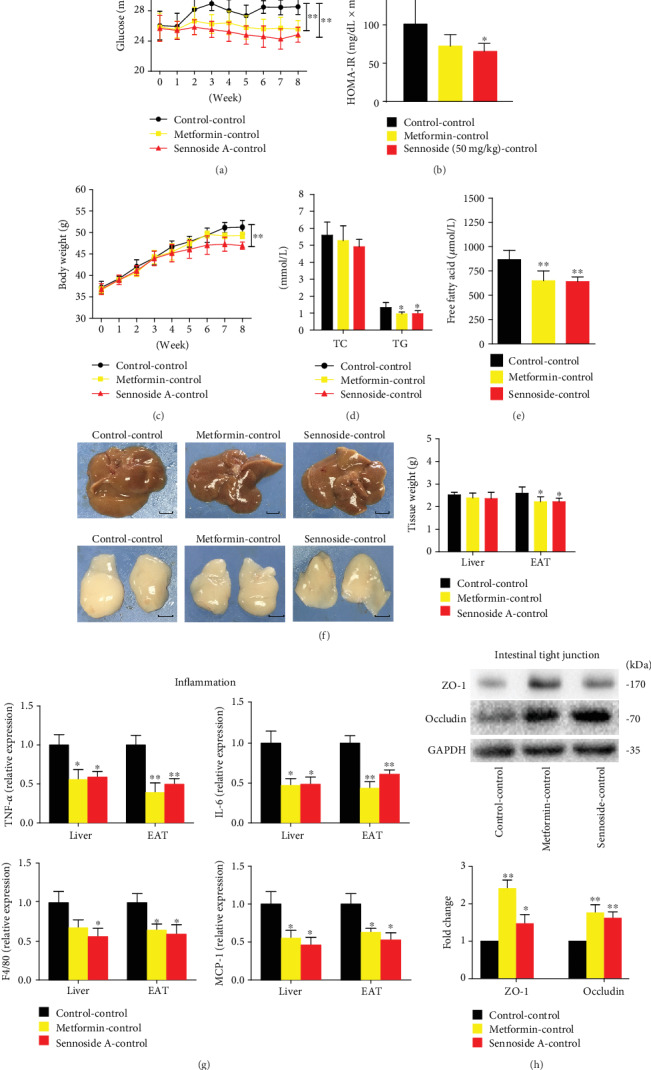
Fecal transplantation of Sennoside A reversed the obesity and T2D-related traits. Mice received a weekly microbiota transplant either from control donor mice (Ctrl-Ctrl) or from Sennoside A (50 mg/kg) donor mice (Sen-Ctrl). Fecal microbiota transplantation (FMT) reversed obesity and blood glucose levels of *db*/*db* mice during the 8-week treatment (*n* = 5 per group). (a) Glucose level, HOMA-IR (b), and body weight (c) of *db*/*db* mice with FMT during 8 weeks. (d) TG and TC of *db*/*db* mice. (e) FFA of *db*/*db* mice. (f) Representative photos of the liver (scale bar: 1 cm) and EAT (scale bar: 1 cm). Weights of the liver and EAT. (g) FMT reduced the expressions of proinflammatory genes in *db*/*db* mice (*n* = 5 per group). Relative mRNA expression levels of proinflammatory genes TNF-*α*, IL-6, F4/80, and MCP-1 in the liver and EAT. (h) FMT prevented a leaky gut as shown in what are representative ileum immunoblots of ZO-1 and occludin.

Similarly, fecal transfer from Sen-treated mice decreased blood glucose level, HOMA-IR index, and body weight in *db*/*db* mice (Figures 6(a)–6(c)). Horizontal fecal transfer from the mice fed with metformin or Sennoside A reduced body weight, TC, TG, FFA, and epididymal fat accumulation compared with those of control mice (Figures 6(d)–6(f)). Fecal transfer from the Sennoside A group exerted the most robust effects on weight gain and fat accumulation.

The mRNA expressions of proinflammatory cytokines also reduced in hepatic and adipose tissues in *db*/*db* mice after the transfer of feces from Sennoside A-treated animals (Figure 6(g)). Furthermore, the expression levels of tight junction proteins in the ileum segment also increased following the transfer of feces from Sennoside A-treated mice (Figure 6(h)).

In order to corroborate that the effects of Sennoside A was interrelated with the gut microbiota, we examined the composition of intestinal bacteria following fecal transfer from Sennoside A-treated mice. In a separate sequencing analysis, a total of 85,620 raw reads and an average of 40, 393 ± 454reads per sample were obtained. Subsequently, a total of 39,819 effective reads were generated. At this sequencing depth, rare phylotypes and most of the diversity were also covered. In comparison with the mice that received feces from control ones, recipient mice (*n* = 5) showed distinct microbiota after FMT from metformin- and Sennoside A-treated ones (Figure S5A). The mice receiving FMT from control ones had the similar Firmicutes-Bacteroidetes ratios and Proteobacteria and Deferribacteres phyla to those of the mice receiving FMT from control ones (Figure S5B). Whereas there were significant changes in the relative abundance of *Akkermansia*, *Mucispirillum*, *Oscillospira*, and *Ruminococcus* by Sennoside A (Figure 3(e)), we examined their levels in FMT experiments. In the recipient mice, the relative abundance of *Mucispirillum*was notably facilitated by Sennoside A-FMT. But the levels of *Akkermansia*, *Oscillospira*, and *Ruminococcus* had been moderately affected by FMT from Sennoside A-treated mice compared with control-treated mice (Figure S5C).

## 4. Discussion

Although previous studies have shown that rhubarb benefited T2D in mice [46, 47], the effects of Sennoside A on blood glucose, gut microbiota, inflammation, and diabetes have not been investigated. Our study revealed that Sennoside A managed to regulate blood glucose so as to alleviate tissue inflammation by modulating gut microbiota composition in T2D mice.

A microbiome with “metabolic disorder” may contribute to obesity and metabolic dysfunction [48, 49], so understanding the regulatory pattern capable of modulating gut microbial composition to a more metabolically favorable state is of major clinical interest. Rhubarb, as the most famous herbal drug in traditional Chinese medicine [10, 16, 50], has been used for centuries to boost health and to lose weight in many developed countries. It may play an important role in attenuating insulin resistance in mice. Jalanka et al. have reported that lavage introduced an instant and substantial change to the intestinal microbiota of patients who received bowel cleansing [51]. We herein postulated that Sennoside A, a vital glycoside in rhubarb, may also be able to reduce the blood glucose in a T2D mouse model by correcting gut dysbiosis.

The microbes, such as certain strains of *Bifidobacterium*, *Lactobacillus*, and *Akkermansia*, are broadly considered to be “beneficial.” Meanwhile, there exists a contrasting assortment of microbes generally considered as directly “harmful” or associated with dysbiosis [52]. In both cases, much of the evidence through which microbes are (often subconsciously) classified as “good” or “bad” is based on observations in highly specific disease conditions, such as diabetes and obesity which were observed in our study. In our study, “good bugs” were negatively correlated with obesity and type 2 diabetes traits, while “bad bugs” were positively correlated with characteristics of obesity and type 2 diabetes. Accordingly, Sennoside A reduced blood glucose in *db*/*db* mice and improved gut microbiota homeostasis. LEfSe that was used to identify (http://huttenhower.sph.harvard.edu/galaxy) differences in taxa composition revealed significantly increased levels of bacterial genuses (good bugs), such as *Odoribacter*, *Turicibacter*, *Akkermansia*, SMB53, and *Mucispirillum* in the Sennoside A group together with decreases in the levels of AF12, *Oscillospira*, and *Ruminococcus* (bad bugs). Spearman's correlation analysis showed that the levels of these bacteria were correlated with obesity and T2D-related traits.

Since the gut microbiota of Sennoside A-treated animals is associated with decreased levels of intestinal Bacteroidetes and increased levels of Firmicutes, these phyla are critical in intestinal permeability and LPS levels [7]. On this basis, we have assumed that Sennoside A may decrease tissue inflammation by modulating gut microbiota that have reduced intestinal permeability and LPS translocation [49]. As expected, Sennoside A has upregulated ZO-1 and occludin protein levels which may bring a protective effect on intestinal barrier function. Meanwhile, the relative mRNA expressions of inflammatory cytokines and LPS in the blood sharply decreased. Nonetheless, the possibility that Sennoside A has directly affected proinflammatory signaling pathways cannot be ruled out.

Blood LPS binds to LPS-binding protein and may interact with various receptors of which TLR 4 activation initiates an extensive intracellular signaling cascade that results in downstream activation of inflammatory signaling pathways and crosstalk with insulin signaling pathways [53]. This explains our results that Sennoside A triggers a reduction in TLR4 protein expression and proinflammatory cytokines such as TNF-*α*, IL-6, and MCP-1. TLR4 signaling pathways can induce the production of proinflammatory cytokines by modulating the activity of NF-*κ*B [54], and the enhanced activation of NF-*κ*B pathways may induce insulin resistance via the dephosphorylation of Akt and GSK-3*β*. All this explains our findings that Sennoside A has also enhanced the phosphorylation of Akt and GSK-3*β*, which may be responsible for increased insulin sensitivity. According to Guadagnini et al.'s article [55], we have further found that Sennoside A could reduce the protein level of TLR2 and hinder the functional activation of JNK.

To confirm the role of gut microbiota in the blood glucose-lowering effect of Sennoside A, we have conducted FMT experiments [56] to evaluate the antidiabetic effects on *db*/*db* mice. Although the antidiabetic effects of FMT from Sennoside A-treated mice cannot be comparable to direct intragastric administration with Sennoside A, the blood glucose level has still reduced significantly. The gut microbiota of Sennoside A-treated mice have been successfully transferred to others for correcting metabolic disorders. We herein have failed to identify a single microbe in the product or to rule out the role of other microbiotas. Also, whether the microbiota is the only contributor or the metabolites of Sennoside A play leading antidiabetic roles remains elusive.

## 5. Conclusion

The results of this study demonstrate that Sennoside A derived from rhubarb exerts significant antidiabetic and antiobese effects on *db*/*db* mice with T2D. Sennoside A can improve microbiota dysbiosis so as to increase intestinal barrier integrity and decrease LPS translocation which result in the alleviation of tissue inflammation and insulin resistance. Therefore, Sennoside A can be used as a microbial regulator to reduce blood glucose, body weight gain, chronic inflammation, and insulin resistance in diabetic individuals. In addition, these data suggest that gut microbiota may be involved in the pathogenesis of T2D and may be ameliorated by Sennoside A.

## Data Availability

The datasets generated and/or analyzed during the current study are available from the corresponding authors on reasonable request.

## Abbreviations

T2D: Type two diabetes
rRNA: Ribosomal RNA
Lepr: Leptin receptor
PCoA: Principal coordinate analysis
OTUs: Operational taxonomic units
TG: Triglyceride
TC: Total cholesterol
TNF-*α*: Tumor necrosis factor-alpha
IL-6: Interleukin-6
mRNA: Messenger RNA
LPS: Lipopolysaccharide
MCP-1: Monocyte chemoattractant protein-1
F4/80: EGF-like module-containing mucin-like hormone receptor-like 1
TLR4: Toll-like receptor 4
IKB-*α*: NF-kappa-B inhibitor alpha
NF-*κ*B: Nuclear factor NF-kappa-B
ZO-1: Zonulaocclidens-1
AKT: Protein kinase B
GSK-3*β*: Glycogen synthase kinase-3*β*
GAPDH: Glyceraldehyde-3-phosphate dehydrogenase
SREBP1c: Sterol response element-binding protein 1c
FASN: Fatty acid synthase
SCD1: Stearoyl-CoA desaturase-1
ACOX1: Acyl-coenzyme A oxidase 1
ACOX2: Acyl-coenzyme A oxidase 2
CPT1*α*: Carnitine palmitoyltransferase I*α*
FFA: Free fatty acid
PBS: Phosphate-buffered solution
qPCR: Quantitative real-time PCR
HE: Hematoxylin-eosin.

## Supplementary Materials

Supplementary Materials Graphical abstract: Sennoside A alleviated T2D and obesity traits by remodeling the gut microbiota in *db*/*db* mice. Figure S1: Sennoside A reduced obesity and glucose homeostasis in *db*/*db* mice. Experiments were performed as in Figure 1. (A) Fasting glucose, (B) body weight gain, (C) TG and TC, and (D) free fatty acids were monitored after a 12-week period of treatments. (E) Representative photos of the liver. Scale bar: 1 cm. (F) Representative HE-stained sections of the liver. Scale bar: 200 *μ*m. Figure S2. Sennoside A reduced the expressions of genes involved in adipocyte differentiation in the liver and epididymal adipose tissues (*n* = 5). Relative mRNA expression levels of lipogenic genes (A) SREBP1c, (B) FASN, and (C) SCD1 as well as lipid oxidation genes (D) ACOX1, (E) ACOX2, and (F) CPT1*α*. Figure S3: (A) differential abundances of the contigs between WT and *db*/*db* mice were calculated for 8 weeks. Bacterial taxonomic profiling at the phylum level of intestinal bacteria from different groups. Experiments were performed as in Figure 2. (B) The rapid increase of food intake, blood glucose, and body weight were delayed after the antibiotic cocktail therapy. (C) The heatmaps show the relative abundance of 33 bacterial genuses (including 11 increased genuses and 20 reduced genuses) significantly altered by 50 mg/kg Sennoside A in comparison with the control group (in *db*/*db* mice). Experiments were performed as in Figure 3(c) and Supplementary dataset S1. Figure S4: the quantification of liver and epididymal adipose tissues immunoblots for TLR4, IkB-*α*, and NF-*κ*B (A and B). Experiments were performed as in Figure 5(b). Representative ileum immunoblots for ZO-1 and occludin in each group (C). Experiments were performed as in Figure 5(f). Insulin signalings in liver (D) and epididymal adipose tissues (E) were analyzed by immunoblotting with antibodies against p-AKT, total AKT, p-GSK3*β*, and total GSK3*β*. GAPDH was used as loading control. Figure S5: mice received a weekly microbiota transplant either from control donor mice (Ctrl-Ctrl) or from Sennoside A (50 mg/kg) donor mice (Sen-Ctrl). Clustering of gut microbiota samples based on next-generation 16S rDNA sequencing analysis. The obesity traits were performed as in Figure 6 by FMT. (A) Plots of weighted UniFrac PCoA was prepared based on OTU abundance matrix. (B) Phylum level analysis of relative abundance of recipients. (C) Relative quantification of *Akkermansia*, *Mucispirillum*, *Oscillospira*, and *Ruminococcus* in different recipient groups. Data are presented as median ± SD (*n* = 5 for recipient groups, respectively). upplementary dataset S1: the Sennoside A-shifted bacterial genuses whose abundance was congruously altered in both Sen versus Ctrl and wild-type versus *db*/*db*.
