# Supplementary material for: Gut Bacteria Selectively Altered by Sennoside A Alleviate Type 2 Diabetes and Obesity Traits
Source: Oxid Med Cell Longev. 2020 Jun 25;2020:2375676. doi: 10.1155/2020/2375676 (PMC7334780; doi:10.1155/2020/2375676)
Supplement: Supplementary Materials — Graphical abstract: Sennoside A alleviated T2D and obesity traits by remodeling the gut microbiota in db/db mice. Figure S1: Sennoside A reduced obesity and glucose homeostasis in db/db mice. Experiments were performed as in Figure 1. (A) Fasting glucose, (B) body weight gain, (C) TG and TC, and (D) free fatty acids were monitored after a 12-week period of treatments. (E) Representative photos of the liver. Scale bar: 1 cm. (F) Representative HE-stained sections of the liver. Scale bar: 200 μm. Figure S2. Sennoside A reduced the expressions of genes involved in adipocyte differentiation in the liver and epididymal adipose tissues (n = 5). Relative mRNA expression levels of lipogenic genes (A) SREBP1c, (B) FASN, and (C) SCD1 as well as lipid oxidation genes (D) ACOX1, (E) ACOX2, and (F) CPT1α. Figure S3: (A) differential abundances of the contigs between WT and db/db mice were calculated for 8 weeks. Bacterial taxonomic profiling at the phylum level of intestinal bacteria from different groups. Experiments were performed as in Figure 2. (B) The rapid increase of food intake, blood glucose, and body weight were delayed after the antibiotic cocktail therapy. (C) The heatmaps show the relative abundance of 33 bacterial genuses (including 11 increased genuses and 20 reduced genuses) significantly altered by 50 mg/kg Sennoside A in comparison with the control group (in db/db mice). Experiments were performed as in Figure 3(c) and Supplementary dataset S1. Figure S4: the quantification of liver and epididymal adipose tissues immunoblots for TLR4, IkB-α, and NF-κB (A and B). Experiments were performed as in Figure 5(b). Representative ileum immunoblots for ZO-1 and occludin in each group (C). Experiments were performed as in Figure 5(f). Insulin signalings in liver (D) and epididymal adipose tissues (E) were analyzed by immunoblotting with antibodies against p-AKT, total AKT, p-GSK3β, and total GSK3β. GAPDH was used as loading control. Figure S5: mice received a weekly micr [file 2375676.f1.zip › mat.2375676.v1 (1).pdf]

Suppementary Materials

Graphical abstract

Sennoside A alleviated T2D and obesity traits by remodeling the gut microbiota in db/db mice.

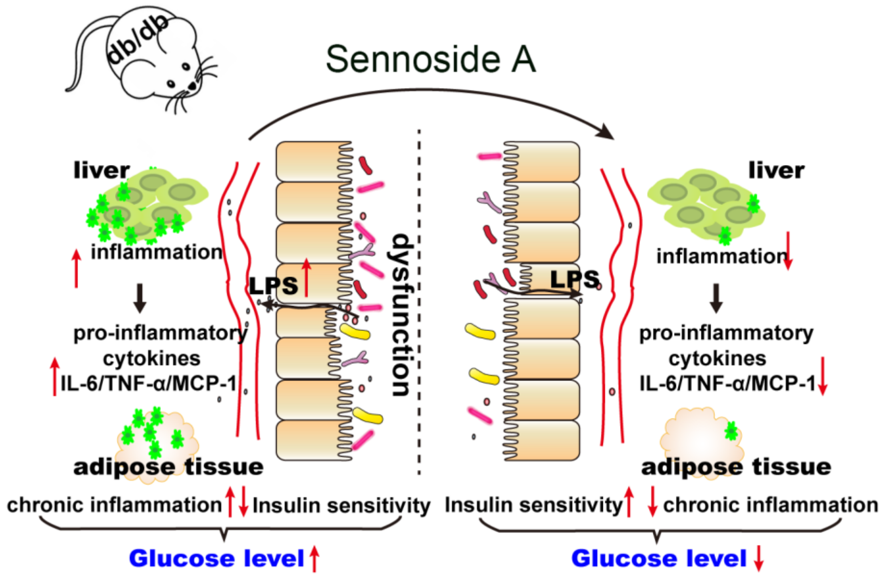

Figure S1

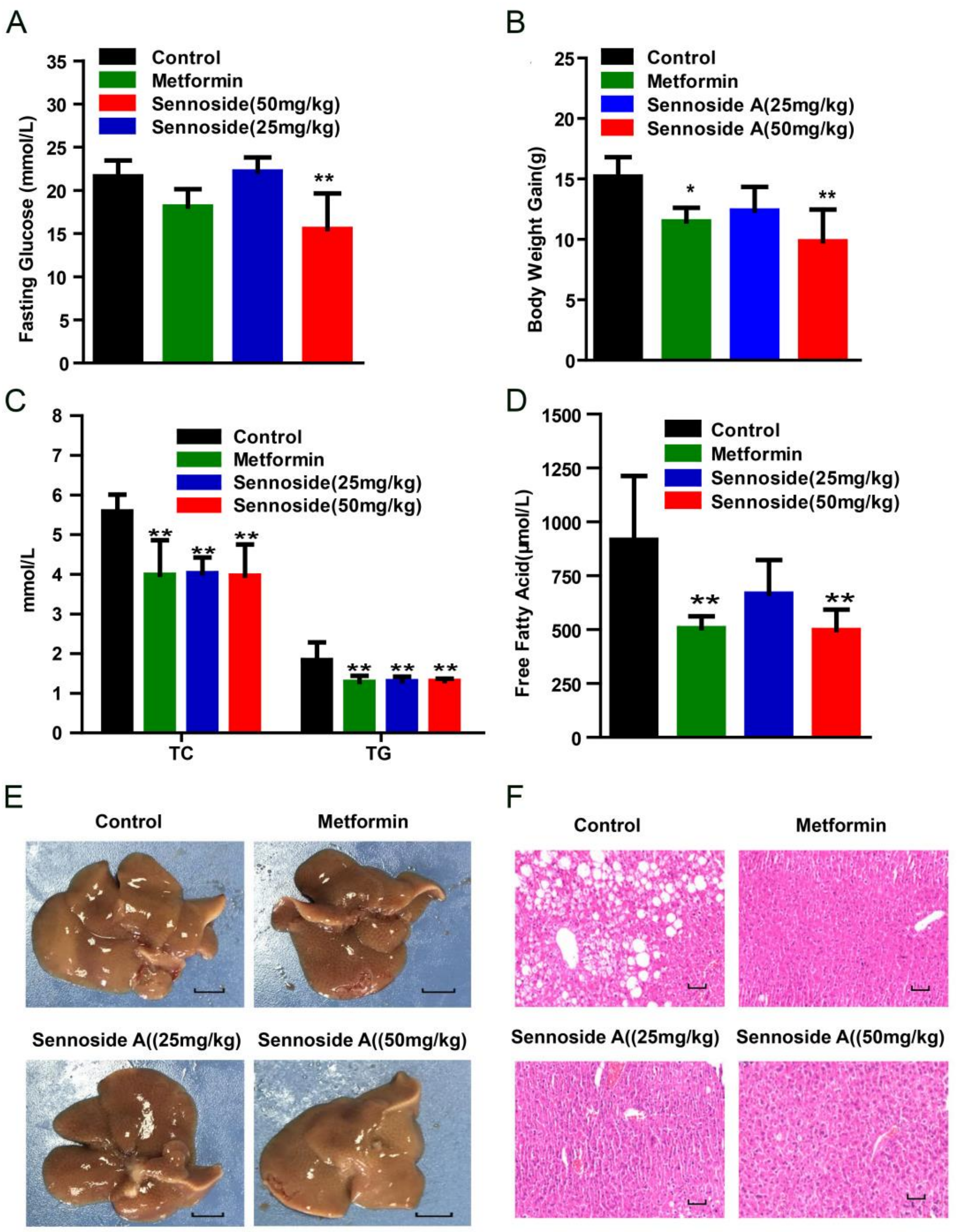

Figure S1. Sennoside A reduced obesity and glucose homeostasis in db/db mice. Experiments were performed as in figure 1. (A) Fasting glucose , (B) body weight gain (C) TG, TC and (D) free fatty acid were monitored after 12-week period of treatments. (E) Representative photos of the liver. Scale bar, 1 cm. (F) Representative HE-stained sections of the liver. Scale bar, 200 μm.

Figure S2

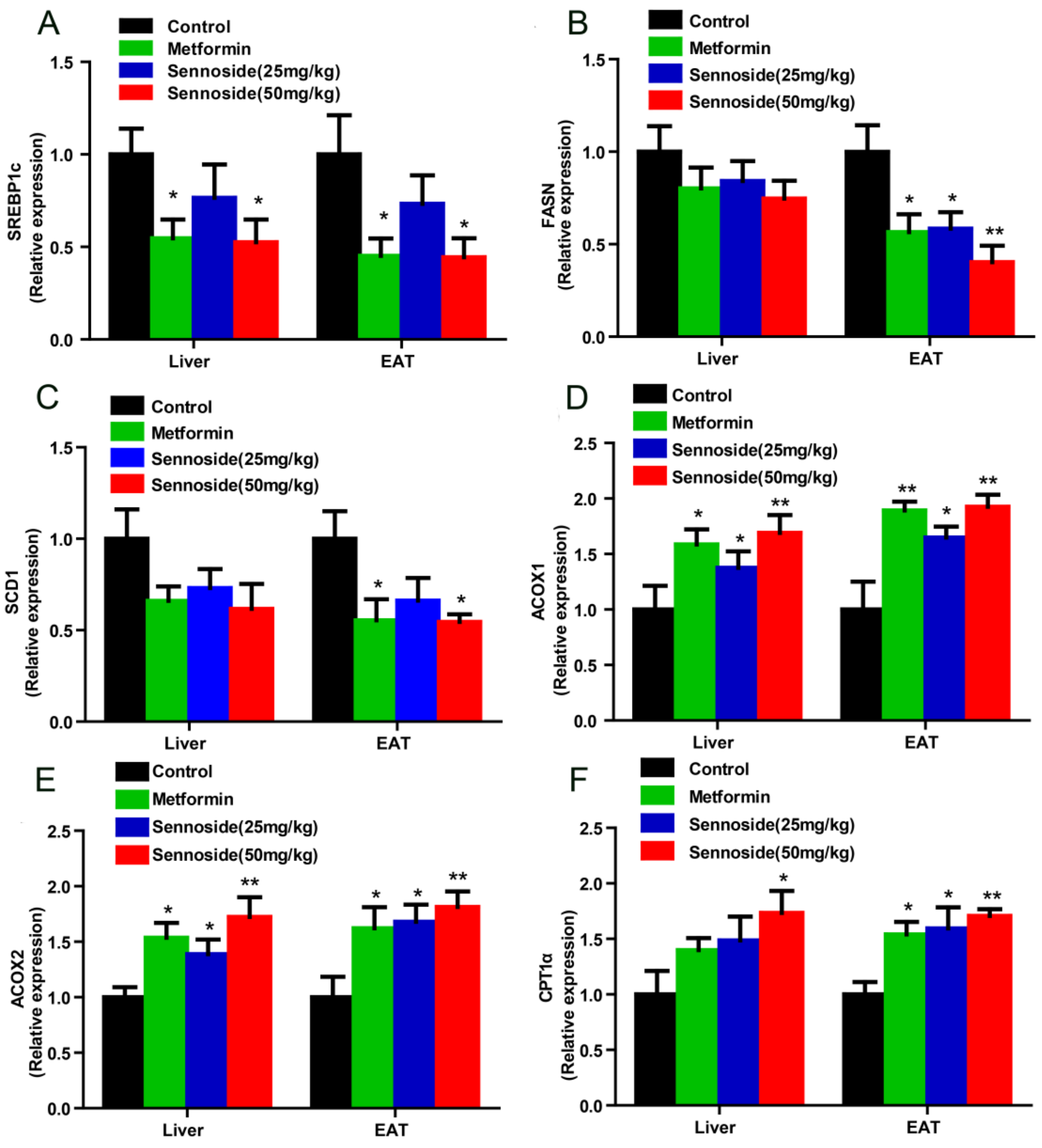

Figure S2. Sennoside A reduced the expressions of genes involved in adipocyte differentiation in liver and epididymal adipose tissues (n=5). Relative mRNA expression levels of lipogenic genes (A) SREBP1c, (B) FASN and (C) SCD1 as well as lipid oxidation genes (D) ACOX1, (E) ACOX2 and (F) CPT1α.

Figure S3

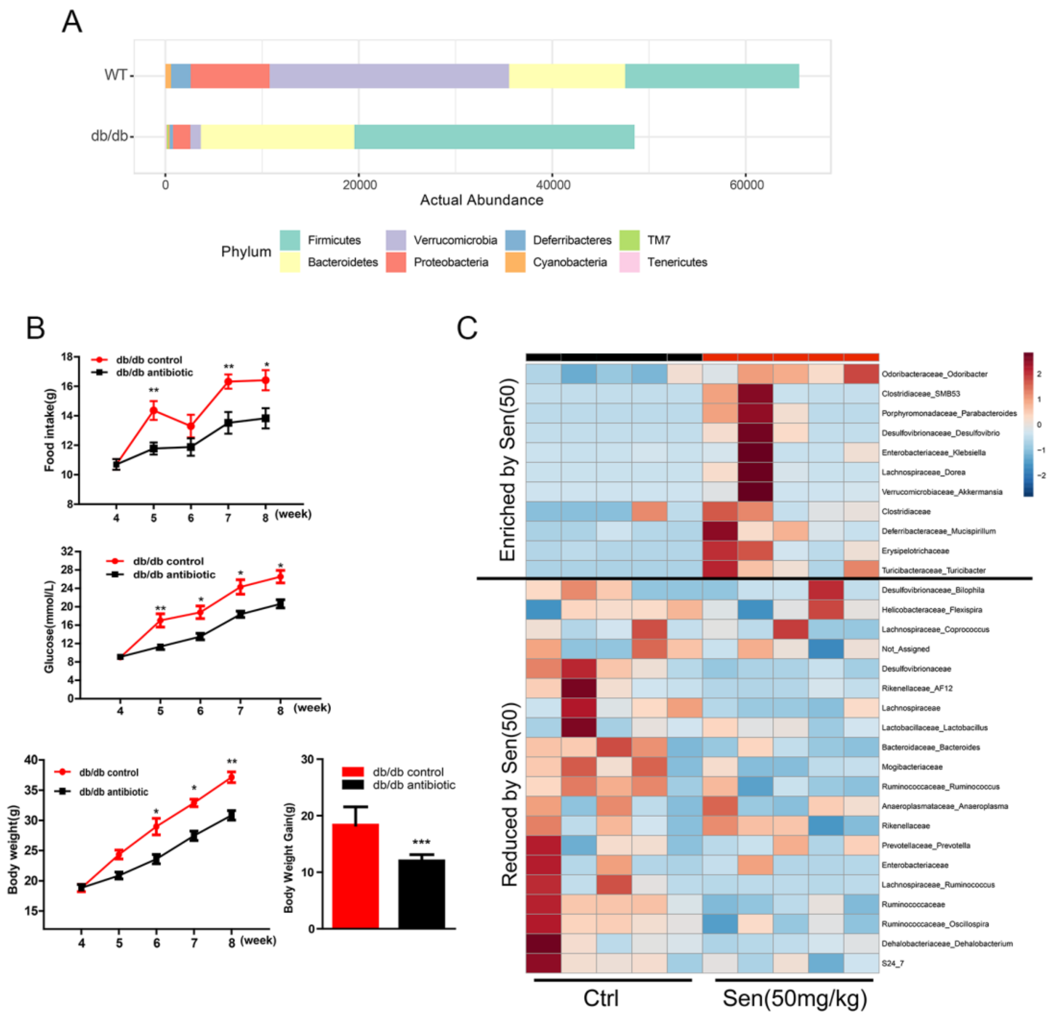

Figure S3. (A)Differential abundances of the contigs between WT and db/db mice were calculated for 8 weeks . Bacterial taxonomic profiling at the phylum level of intestinal bacteria from different groups. Experiments were performed as in figure 2. (B) The rapid increasement of food intake, blood glucose and body weight were delayed after the antibiotic cocktail therapy. (C) The heatmaps show the relative abundance of 33 bacterial genuses (including 11 increased genuses and 20 reduced genuses) significantly altered by 50mg/kg Sennoside A in comparison with the control group (in db/db mice). Experiments were performed as in figure 3C and supplementary dataset S1.

Figure S4

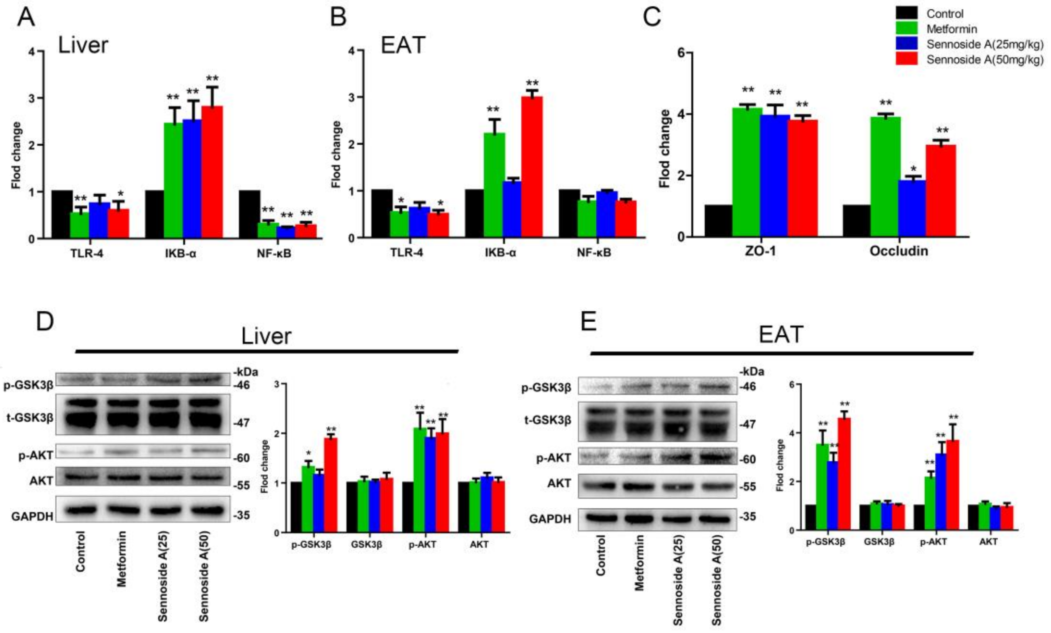

Figure S4. The quantification of liver and epididymal adipose tissues immunoblots for TLR4, IkB-α and NF-κB (A&B). Experiments were performed as in figure 5B. Representative ileum immunoblots for ZO-1 and occludin in each group (C). Experiments were performed as in figure 5F. Insulin signalings in liver (D) and epididymal adipose tissues (E) were analyzed by immunoblotting with antibodies against p-AKT, total AKT, p-GSK3β and total GSK3β. GAPDH was used as loading control.

Figure S5

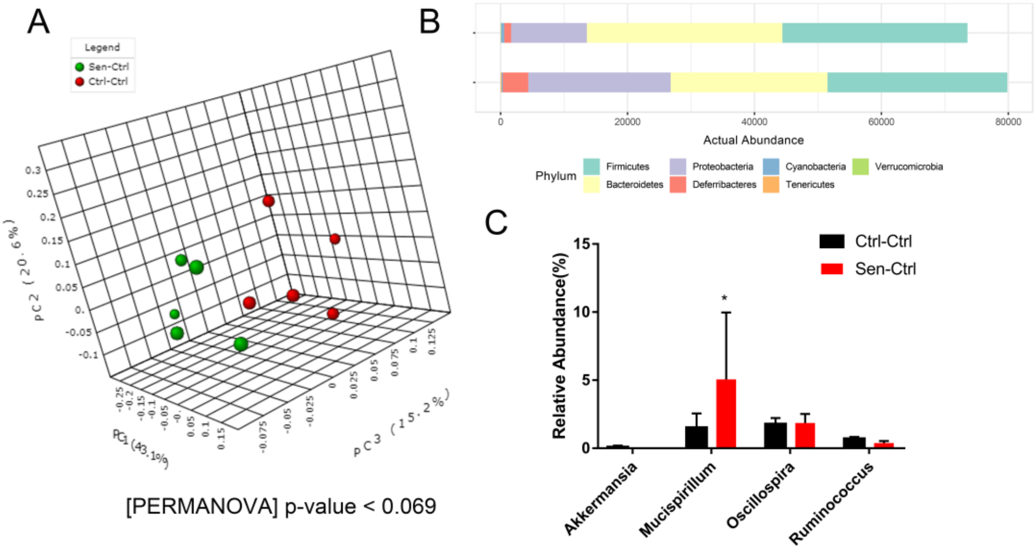

Figure S5. Mice received a weekly microbiota transplant either from control donor mice (Ctrl-Ctrl) or from Sennoside A (50mg/kg) donor mice (Sen-Ctrl). Clustering of gut microbiota samples based on next-generation 16S rDNA sequencing analysis. The obesity traits were performed as in figure 6 by FMT. (A) Plots of weighted UniFrac PCoA was prepared based on OTU abundance matrix. (B) Phylum level analysis of relative abundance of recipients. (C) Relative quantification of Akkermansia, Mucispirillum, Oscillospira and Ruminococcus in different recipient groups. Data are presented as median±SD (n=5 for recipient groups, respectively)

Supplementary dataset S1 : The Sennoside A-shited bacterial genuses whose abundance was congruously altered in both Sen versus Ctrl and wild-typed versus db/db.
